# Gluten and Autism Spectrum Disorder

**DOI:** 10.3390/nu13020572

**Published:** 2021-02-09

**Authors:** Iain D. Croall, Nigel Hoggard, Marios Hadjivassiliou

**Affiliations:** 1 Department of Infection, Immunity & Cardiovascular Disease, University of Sheffield/INSIGENO, Sheffield S10 2JF, UK; n.hoggard@sheffield.ac.uk; 2 Academic Departments of Neurosciences and Neuroradiology, Sheffield Teaching Hospitals NHS Trust, Sheffield S10 2JF, UK; m.hadjivassiliou@sheffield.ac.uk

**Keywords:** autism spectrum disorder, gluten sensitivity, celiac disease, coeliac disease, review

## Abstract

An expanding body of literature is examining connections between Autism Spectrum Disorder (ASD) and dietary interventions. While a number of specialist diets have been suggested as beneficial in ASD, gluten has received particularly close attention as a potentially exacerbating factor. Reports exist suggesting a beneficial effect of the gluten-free diet (GFD) in ameliorating behavioural and intellectual problems associated with ASD, while epidemiological research has also shown a comorbidity between ASD and coeliac disease. However, both caregivers and clinicians have expressed an uncertainty of the value of people with ASD going gluten-free, and as the GFD otherwise receives considerable public attention a discussion which focuses specifically on the interaction between ASD and gluten is warranted. In this review we discuss the historical context of ASD and gluten-related studies, and expand this to include an overview of epidemiological links, hypotheses of shared pathological mechanisms, and ultimately the evidence around the use and adoption of the GFD in people with ASD.

## 1. Motivation and Literature Search Methods

Autism Spectrum Disorder (ASD) is a neurodevelopmental disorder characterised primarily by deficits in social communication and restricted/repetitive patterns of behaviour (DSM-5) [1]. As the name implies its phenotype exists on a spectrum, and overall it is estimated to affect as many as one in 69 children [2]. Interest in the use of specialist diets in ASD is increasing, as a way to alleviate its behavioural and intellectual outcomes. Though many dietary interventions have been suggested, the gluten-free diet (GFD) is among the most notable. Clinically, the GFD is well recognised as the primary treatment for patients with a gluten-related disorder. The most prominent of these is coeliac disease (CD) which is predominantly expressed as a gastrointestinal (GI) condition. However, physiological sensitivity to gluten is known to exist in other forms. These include other immune-mediated disorders (e.g., dermatitis herpetiformis and gluten ataxia), allergic reactions (wheat allergy), and non-coeliac gluten sensitivity (a condition characterized by self-reported gastrointestinal and extra-intestinal symptoms subjectively improving upon a GFD in subjects in whom other major organic gluten related disorders have been excluded) [3]. The clinical utility of the GFD outside of these contexts is debatable, and elsewhere its adoption within the general public as a sometimes “fad” diet heightens such scrutiny [4]. However, generally increased rates of GI problems have been reported in people with ASD, as has evidence of an apparent comorbidity between ASD and CD specifically. As adoption rates of specialty diets which include a gluten-free component are very high in ASD we were motivated to conduct a literature review which focused specifically on the interaction between ASD and gluten.

Searches were made on Pubmed on the 12th of November 2020. Terms included:

- Autism coeliac
- Autism celiac
- Autism gluten
- Autism wheat
- Autistic coeliac
- Autistic celiac
- Autistic gluten
- Autistic wheat

**Figure 1:**
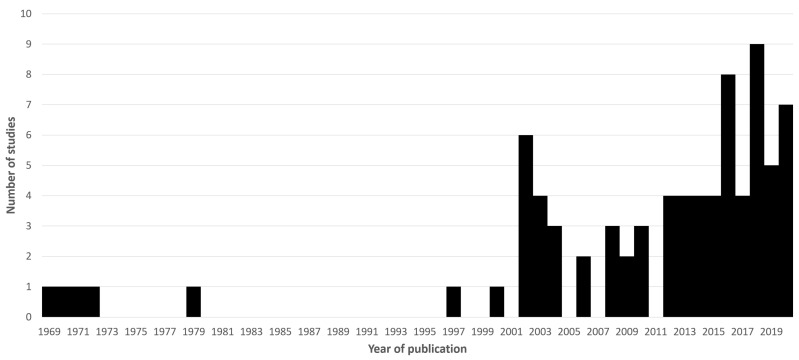
A histogram of eligible papers found in the primary pubmed search according to their year of publication.

These terms were designed to capture a range of relevant terminology, for example “autism spectrum disorder” or “gluten free diet” would each be picked up by these. Regional variation in spelling of coeliac/celiac would also be accounted for. This returned 237 unique articles. The abstracts of all of these were read to determine eligibility for inclusion in the main review. Criteria for this were that the paper must be original research (i.e., not another review or systematic review) which in some way directly explored links between ASD and GRDs, the use of the GFD in ASD, or any other relevant interaction between ASD and gluten. Case reports were excluded, as were papers where the main article was not available in English. Seventy-nine articles were deemed eligible for inclusion. These were read, and any additional relevant citations found through this were added for discussion. Figure 1 shows these included papers according to their year of publication.

Throughout the study of the literature, common research methods/sub-topics were noted. The remainder of this review synthesises these papers according to those themes. Additional literature was searched for and referenced where necessary to elucidate on a relevant key concept which was not adequately covered by the initial searches.

## 2. Historical Context

The first observation of a possible link between gluten and ASD was reported in 1969 by Goodwin & Goodwin [5], who noted in a cohort of 65 children with ASD that one 6 year old boy also had CD. This child’s subsequent treatment with a GFD appeared to improve outcomes relating to his ASD. It is relevant to note that at that time the prevalence of CD was considered to be far less than the 1-in-100 that it is sometimes reported as today [6], largely due to the lack of effective diagnostic methods such as serological testing. In a commentary piece on the then-emerging topic, Dohan references in their 1970 paper [7] that CD is thought to affect approximately 1 in 3000 people, making suspicion after finding it in one of a cohort of 65 children with ASD understandable. Another point of interest emerges from this paper, which was purely focused on links between CD and schizophrenia but in which Dohan also found it relevant to include an anecdotal report of increased CD rates in ASD groups (“at least 2 coeliac patients among 140 severely autistic children”). While limited phenotypic similarities between schizophrenia and ASD are still discussed now, initial characterisation of the conditions involved such overlap that full clinical separation did not occur until 1980 with the publication of the DSM-III [8]. To a modern reader, this explains why there are a number of early articles which combine ASD with schizophrenia and otherwise draw potentially confusing links between the two.

Goodwin et al. published another study in 1971 [9] which is arguably the first trial investigating how gluten modified behaviour in a cohort of children with ASD. Also included were controls and a group of participants with schizophrenia for further comparison. Here, the participants followed the “sprue diet” (GFD) for a single day, in which they were also given a cherry drink which had added to it either gliadin or a placebo (sugar). They were then subsequently monitored and tested via investigation of blood counts and electrophysiological recordings by trans cephalic direct current. Although the authors note some findings, these predominantly focus on differences which appear to separate ASD and schizophrenia participants, providing only one comment on the effect of gliadin where it appeared to reduce plasma cortisol levels. However, this was observed across both control and ASD participants, and when coupled with the small sample sizes (the ASD group had 9 participants for that portion of the results) and extremely short period of dieting, it is difficult to extrapolate any meaningful conclusions. Further studies in the 1970’s included a comparison of serum alpha-1-antitrypsin levels between children with ASD vs. children with CD [10] (finding them comparably abnormal and suggesting a shared pathology), an experiment published in a book [11] describing 72 patients with ASD in whom CD was diagnosed (without biopsy) in 8, and another trial of gluten in eight children with ASD who already followed a GFD and were purportedly better for it [12]. This latter study saw the participants stop their diet to undergo a gluten challenge for 1 month, hypothesising this would worsen their phenotype, but finding no change in bodily measurements (weight, bowel habit etc.) or behaviour (measured by parental reports and observation from a specialist paediatrician).

To summarise, early interest in the topic was driven by essentially anecdotal reports of apparently comorbid cases of CD with ASD. Direct experimentation of this resulted in largely negative findings. These studies had small samples sizes and, when viewed with a contemporary lens, suffered from experimental designs and measurement techniques which would now be considered extremely insensitive in targeting relevant outcomes. Following these papers, little relevant research activity appeared until the mid 1990’s when the topic appeared to become more popular once again.

## 3. Gastrointestinal Symptoms in ASD

A heightened rate of gastrointestinal (GI) symptoms in people with ASD is well documented. While this particular topic in its entirety falls outside of the scope of the present systematic review, the observation of these symptoms is a major motivator for gluten-specific research. Relevant studies from the review are therefore included here, as well as other key literature.

In 2014 Chaidez et al. [13] conducted a large study in which 499 children with ASD were compared to typically-developing (TD) children (*N* = 324) and children with developmental delay (*N* = 137) in terms of GI symptoms measured by 10 Likert scales (abdominal pain, constipation etc.). After controlling for age, sex, maternal education and medications which may lead to GI side effects, children with ASD had significantly heightened odds ratios (OR) compared to controls for 8 outcomes, the lowest being 3.14 (abdominal pain) and the highest being 8.61 (sensitivity to foods). The children with ASD and developmental delay were not significantly different from one another.

This is one of a number of similar studies who’s findings are supported by meta-analyses; a pubmed search of “gastrointestinal autism” found the most recent meta-analysis was performed in 2014 [14]. This included 15 studies which gave a combined sample of 2215 children with ASD in which four variables were included; general GI concerns, diarrhoea, constipation and abdominal pain. Each of these was found to be significantly more prevalent in the ASD group compared to TD children, with overall OR’s of 4.42, 3.63, 3.86 and 2.45 respectively.

Relevant studies from the current review include a report [15] of a higher frequency of constipation in children with ASD, a study [16] which found children with ASD and regression more often had abnormal stool than those without regression, and another experiment [17] which found GI symptoms to be more common in children and adolescents with ASD than in TD controls, and for these symptoms to be weakly correlated to behavioural measures. Correlations such as these have been documented elsewhere [18,19,20]. These studies reference the often non-specific nature of GI symptoms with one explaining that “a GI pathology specific to ASD had not been established” (Babinska et al., 2020 [17]). Regardless, a notable body of literature has investigated for a comorbidity between ASD and specific GI conditions, often finding significant results.

## 4. The Co-Morbidity between ASD and CD

Following early literature from the 1970’s, the first study to investigate for an increased rate of CD in ASD was Pavone et al. in 1997 [21]. Pavone examined a cohort of children with ASD (*N* = 11) to detect the rate of CD (by antibody and ultimately biopsy testing), and similarly a cohort of children with CD (*N* = 120) to detect the rate of those with features of ASD (as reported by parents and according to the DSM III-R). None of the children with ASD had biopsy-proven CD, while none of the children with CD met criteria for a full ASD diagnosis (though a limited few did show isolated features). This was therefore overall a negative study, but the limitations of examining in such small cohorts as 11 are evident.

Since then, a limited number of large epidemiological studies have been conducted which generally do show an effect indicating CD and ASD to be comorbid to one another. One of 2009 [22] which focused specifically on comorbidities to ASD within parental medical history, used the Danish Civil Registration System to identify all children born between 1993 and 2004 with ASD (*N* = 3325). Here, maternal history of CD led to a significant, overall incidence rate ratio (IRR) of 2.97 in terms of the child having ASD. Other studies examining comorbidities within the same participant have also found significant results while using medical databases. A 2017 study [23] examined for the risk of psychiatric sequalae in children with CD (*N* = 10,903), finding a hazard ratio (HR) of 1.5 (univariate analysis) of being diagnosed with ASD after their CD diagnosis but before the age of 18 (adult data was not included). The same research group has replicated this more recently [24] with a larger cohort of people diagnosed with CD while a child (*N* = 19,189), but which this time did also include psychiatric diagnoses obtained after 18. Here, the HR of developing ASD was 1.47, the highest of all disorders included in analyses.

These papers do however contrast an earlier study, again by the same research group [25], which examined specifically for the likelihood of an ASD diagnosis *preceding* a CD diagnosis in children and adults with CD (*N* = 26,995). This OR was non-significant, though potentially of interest was a finding wherein previous ASD was still associated with an increased risk of having normal mucosa on biopsy, but positive CD serological test results (tissue transglutaminase; TTG, endomysial; EMA or gliadin; AGA antibodies, reported as a single grouping). While the immediate clinical implication of positivity to these antibodies varies, i.e., TTG/EMA positivity indicates CD with high sensitivity/specificity while many generally-healthy individuals may exhibit AGA positivity, it should be highlighted that within the study of wider “gluten sensitivity” heightened rates of any of these may be considered pathologically-relevant when compared to an appropriate “control” such as in this discussed study. This is therefore an important study as it highlights the link between serological markers of gluten sensitivity and ASD in the absence of enteropathy.

Other prevalence research has been conducted with less stringent diagnostic criteria and/or smaller sample sizes, finding mixed results. Studies with significant findings include Calderoni et al. [26] who examined a cohort of children with ASD (*N* = 382) and found the rate of CD within the sample was 2.62%, although it should be noted this sometimes relied only on a positive serological (TTG/EMA) result and formal CD diagnosis was not always confirmed. Valicenti-McDermott et al. [16] found an increased family history of CD and/or inflammatory bowel disease in children with ASD who also exhibited regression (*N* = 24), compared to children with ASD without regression (*N* = 71). In a letter to the editor, Barcia et al. [27] report an experiment where of 91 “randomly selected” children with ASD, 4 had “biopsy-proven” CD (the authors reference diagnostic guidelines for diagnosis where Marsh grade 3 denotes CD). This is a rate of 4.4% which is considerably higher than might be expected. Mazzone et al. [28] found in a cohort of 100 children with CD that 2 had ASD (while none of a control group did); whether this is a positive result or not is arguable.

Studies with negative findings include Alabaf et al. [29] who via parental reporting of 91 children with ASD did not find an association with CD (not reported but this was measured, implying a negative finding). Juneja et al. [30] screened children with ASD (*N* = 150) for CD defined by IgA TTG testing, finding no positive tests. In 2012, Batista et al. [31] examined children and adolescents with either ASD (*N* = 147) or biopsy-proven CD (Marsh grade 3, *N* = 211) for the rate of the other. The ASD group was found to be entirely negative for CD (although one subject did have a weakly-positive TTG result with negative EMA), while two cases of ASD were found in the CD group; this was concluded to not be above chance. Zelnik et al. [32] examined a cohort of CD patients (*N* = 111) for a range of neurological outcomes including ASD, although as this was reported mixed in with other learning disabilities and ADHD (which overall affected 20.7% of the group) how common ASD specifically was is not known. Finally, Black et al. [33] examined medical records to identify 96 children with ASD and reported on all diagnosed gastrointestinal comorbidities, failing to identify any CD cases.

A recent meta-analysis [34] combined some of the above studies (where eligible) to find a significant odds ratio of 1.53 in terms of CD patients having ASD, but a non-significant likelihood of ASD participants having CD. Overall therefore, the majority of studies relevant to the question of comorbidity have used variable sample sizes and diagnostic methods, making definitive conclusions difficult in most individual instances. However, the strongest powered are undoubtedly those from Sweden which studied large cohorts and established an increased risk of a subsequent ASD diagnosis in people with CD, therefore showing a convincing comorbidity. This is further supported by the meta-analysis finding, which showed the same. Studies which examine the “reverse” of this, where initial ASD features may increase risk of subsequent CD, have led to more negative findings however do demonstrate an association with the development of gluten antibodies in the absence of clinical CD. It should also be highlighted that a very large epidemiological study which principally studies an ASD cohort for the rate of CD is absent, which may introduce a sampling bias when interpreting the findings of this overall field. Overall therefore, ASD does appear comorbid to CD, and while an increased risk of CD in ASD is not currently supported a suspicion of ASD being linked to subsequent, immune-mediated “gluten sensitivity” may be warranted.

## 5. Hypothetical Mechanisms of Action

With a comorbidity between ASD and CD established, a natural question is of what shared pathophysiology may drive these associations. Further, as non-specific GI symptoms are also seen to be generally more prevalent in ASD and that gluten sensitivity is increasingly understood to be a spectrum that extends beyond the clinical criteria for CD specifically, it is important to consider any mechanism of action between gluten and ASD.

From an early point in the literature, hypotheses have frequently related in some way to heightened autoimmunity in ASD. While a predisposition towards autoimmunity was noted as early as 1971 [35], enquiries of this nature gathered pace after a key publication in 2001 [36] which showed children with ASD (with regression) to have increased markers of innate and adaptive immune response (TNF-A, cytokines etc.). This was investigated at the time partially in response to parental reports of children with ASD suffering apparently high rates of reactions to dietary irritants, and this autoimmune phenotype was subsequently hypothesised to be part of the aetiology of ASD. The authors presented this idea in terms of environmental stimuli triggering an immune response which exacerbates ASD features, and in the specific case of their study hypothesised it may stimulate regression.

In the early 2000’s a series of publications by Vojdani et al. [37,38,39] built on this evidence by focusing on more specific dietary triggers and hypothetical knock-on effects they would lead to in terms of molecular pathways. Here, focusing mainly on gliadin (a gluten-specific protein) and casein (a protein in dairy products), it was demonstrated that children with ASD have high rates of antibodies against these (i.e., anti-gliadin and anti-casein) as well as antibodies against DPP4, a digestive enzyme. DPP-4 is important in the processing of gliadin. Initially, gliadin is degraded into various peptides which include gliadinomorphin-7 [40], an immune reactive substance with “opioid activity”, i.e., which stimulates opioid receptors in the body [41]. Further degradation of gliadinomorphin-7 is therefore required, which is where DPP4 functions by cleaving such peptides [42]. As Vojdani et al. reported, the existence of anti-DPP4 would hypothetically reduce the amount of circulating DPP4, increasing the abundance of gliadinomorphin-7 and the likelihood of downstream, opioid-like effects. It should be highlighted that casein and other dietary peptides are similarly degraded to intermediary substances with opioid properties (e.g., casomorphin [43]), and together these potentially harmful peptides have been termed “exorphins” [44].

Stimulation of the opioid system has been studied in the context of ASD features. An early proponent of this link, Panksepp outlined a theory in 1979 [45] (based largely on his earlier animal model experiments) wherein excess opioid activity may lead to the decreased social behaviour seen in ASD. This theory has persisted until today, with numerous publications concerned with evidence of opioid overactivity in people with ASD. Animal studies have continued to show the importance of a balanced opioid system in maintaining social behaviours which are similar to those impacted in ASD, while experiments investigating for levels of relevant, opioid-like peptides in the sera, CSF or urine of people with ASD have generally shown raised titres albeit with some notable exceptions where decreases have been reported [46]. Indeed, measurement of urine peptides has become a common tool in this field, with high levels being seen as an indication of insufficient digestion of food which may lead to excess exorphins [47].

An alternative theory has focused on the role that oxidative stress may play in ASD, which may lead to a state of inflammation in the brain. It has for example been reported that people with ASD have an impaired antioxidant defence in the cerebellum [48], while problems metabolising nitrous oxide (which may lead to increased oxidative stress [49]) has also been proposed as driver of ASD pathophysiology [50]. This holds a relevance to gluten sensitivity, where increased oxidative stress has also been demonstrated, for example as triggered by gliadin [51] or as demonstrated generally by raised markers of oxidative stress across untreated children with CD [52].

Studies have also noted the potential for shared genetic predisposition. One recent paper by Bennabi et al. [53] compared genotyping data between ASD and control cohorts, finding that the haplotype HLA-DRB1*11-DQB1*07 was more common in the ASD group, with this being more prevalent still in those ASD patients with the most pronounced behavioural symptoms. A different haplotype (HLA-DRB1*17-DQB1*02) was conversely more common in the control group. The *07 haplotype was therefore concluded to be potentially causative and the *02 one protective. Of relevance is that the *07 haplotype is additionally recognised as associated with CD, leading to a suggestion that there may be a sub-group of people with ASD holding a genetic risk for both [54]. However, other genetic research has produced negative findings, such as a meta-analysis of genome-wide association studies [55] which did identify regions associated with ASD but noted only overlap between these and schizophrenia.

The potential of reactivity of antibodies to gluten products should be discussed. Antibodies against tissue-transglutaminase (TTG) have very high sensitivity and specificity in diagnosing CD, meaning that as a comorbidity has been demonstrated they may have a relevance in ASD pathology. These antibodies have been reported to lead to apoptosis of neuroblast cells in vitro [56]. Other antibodies which may indicate gluten sensitivity but not CD specifically include transglutaminase 6 (TG6) antibodies, which have been indicated in the diagnosis of gluten ataxia [57] (where the cerebellum is the primary site of damage), with this supported by animal research showing TG6 to be distributed throughout the central nervous system including brain regions such as the cerebellum and thalamus [58]. TG6 antibodies have been reported at a rate of 4.4% in a group of 77 children with ASD [59]; this experiment lacked a control group and as this is a relatively novel marker it is difficult to evaluate if this is abnormal. Finally, gliadin antibodies have been shown to react with brain blood vessel structures [60], show cross-reactivity with neuronal synapsin 1 [61], and to be associated with rates of depression in people with CD and healthy controls [62]. Gliadin antibodies have been measured across a number of studies in ASD, frequently finding them to be raised. Those found in the current review are summarised in Table 1.

The potential for antibodies and other irritants to travel from the gut to the brain is raised by a number of studies which have demonstrated generally inflamed/abnormal intestinal findings in ASD [68,69,70,71], and others showing compromised intestinal permeability specifically [66,72]. Indeed, one other publication [73] examined gene and protein expression of brain and intestinal tissue of human ASD subjects, finding evidence of impaired intestinal permeability in combination with altered blood-brain barrier integrity. It should be noted that not all studies support an impacted intestinal permeability [59,74], but nonetheless these phenomena raise the possibility of a gut-brain axis interaction being relevant in ASD. Here, a negative feedback loop between the brain and the gut would lead to exacerbation of both neurological and GI outcomes. Arguably most studies which have investigated how gluten can impact people with ASD may fall within this broader concept, where irritants enter the bloodstream from the gut to cause downstream negative consequences for the brain. A loop may be completed if the effect on the brain leads to alterations of behaviour and appetite which may maintain or exacerbate the cycle [75].

In summary, pathological interactions between ASD and gluten have focused on opioid activity from improperly digested gluten products, inflammation caused by oxidative stress and/or reactivity with anti-gluten antibodies, and some indications of shared genetic factors. These hypotheses provide some explanation for the previously discussed comorbidity between ASD and CD and also make it appear reasonable that gluten may exacerbate bodily stress in other groups of people with ASD who do not have CD. However, it remains unclear to what extent ASD populations and sub-populations are affected, and to what degree these findings represent a unique interaction with gluten specifically or are a consequence of a generally-raised autoimmune profile in ASD.

## 6. Trials of the GFD in ASD

Establishing that gluten is potentially harmful for people with ASD leads to the question of if a GFD would then bring any benefits. After the early studies of the 1970’s, the first trial which involved gluten in any capacity was conducted in 1990 and is detailed in two publications [76,77] by Knivsberg et al. Here, fifteen children with ASD in combination with abnormal urine peptide results engaged with a gluten and casein-free diet (GCFD) for four years. Measurements were generally taken at baseline, one year and four year time points, and included scales which characterised psychotic behaviour in children, psycholinguistic ability and fluid intelligence. However, this data was not all collected consistently (e.g., the psychotic behaviour measurements were not made at 4 years), and authors note variable dietary success between the children. Regardless, significant findings suggested improvement across multiple outcomes, including normalisation of urine peptides. The 1990’s saw one other trial [78], the primary analyses of which concerned a group of 22 children with mixed spectrum disorders (the most common being ASD) who undertook a GFD for 5 months. Other groups were also examined, e.g., children with ASD already on a GFD took a gluten challenge, although these sample sizes were very small. Similar to the Knivsberg study, improvement in behavioural outcomes was noted in response to the GFD although no change in urinary peptide levels were seen.

The first randomised trial was conducted in 2002 [79]. Here, 20 children with ASD and abnormal urinary peptides were randomised into parallel groups to receive either the GCFD or a regular diet for 12 months. Following this, improvements were noted across behavioural and intellectual outcomes. A number of randomised trials have been conducted since and those that utilise an intervention that in any way involves gluten are summarised in Table 2.

Examining this literature reveals a very mixed picture of findings. Of the 13 RCT’s found in the current review, improvements of some kind were noted in 6 [79,82,83,84,85,90], no findings were observed in another 6 [80,81,86,88,89,91], while a worsening of GI symptoms (in response to the GCFD) was observed in one study [87]. All 6 studies which noted a positive effect from the interventional diet included improvements in intellectual/behavioural outcomes, and sometimes also in physiological measurements (e.g., GI symptoms).

Consolidation of these studies is difficult even beyond the mixed findings. One immediate observation is that the exact dietary intervention employed is variable. This increases heterogeneity between studies and makes commenting on the effect of gluten specifically impossible in most cases. Only 3 of the 13 trials had a group design which in some way tested the GFD in isolation; one of these reported improvements in outcomes [85]. Others typically focus on the GCFD (the most investigated of all interventions), while some use unique interventions such as Grimaldi et al. [82] who primarily tested a probiotic mixture (in combination with the GCFD). The study by Adams et al. [83] is also notable for the approach of sequentially accumulating interventions over a year which included the likes of dietary supplementation and epsom salt baths, with the GCFD being added at day 210.

The majority of studies are also unblinded (8 of 13) which raises a risk of placebo/nocebo effects. Some trials which are blinded use as a placebo gluten-free versions of food (bread etc.) given to participants on the assumption that they will not be able to tell the difference, meaning that a degree of skepticism is warranted even for those with such an experimental design. Authors of non-blinded trials that achieve significant results acknowledge this limitation but highlight the practical difficulty of effective blinding for a GFD over a long period of time, or blinding of the other mixed interventions employed. This often leads to varying levels of “blindedness” within a trial, depending on the specific intervention/outcome examined. For example Adams et al. [83] write “A strength of the study is that it was a randomized, controlled study, but a major limitation of this study is that implementation of a healthy, HGCSF (healthy/gluten/casein/soy-free diet) does not allow blinding of participants. The RIAS evaluation was single-blinded, and the CARS and SAS-Pro were semi-blinded (the evaluators were blinded, the participants were not), so those results are fairly robust. The parent evaluations certainly are subject to some placebo-effect but provide an upper-bound on possible benefits. The laboratory measurements were conducted in a blinded manner, so those results should be reliable.”

Studies variably do or do not use dietary “run-in” periods, which would be generally advisable to account for delays in physiological adjustment between different regimens when taking experimental measurements. Some trials are conducted over very short timeframes, such as Navarro et al. [88] which ran for 4 weeks or Pusponegoro et al. [87] which ran for 1 week. The implication of this will vary depending on the outcomes measured, but regarding gluten it is for example known that resolution of symptoms due to gluten exposure can take a number of weeks in patients with CD [92], while achieving gliadin antibody negativity can take 6 months or longer [93]. This emphasises the need for long term trials if adequate time is to be given for changes to be captured. In terms of assessing change, the measurement scales used are also scarcely replicated between studies. The majority of RCTs employ a set of tools which are unique to that particular trial, further complicating comparisons or synthesis of findings. An effort to arrive at an agreed-upon set of outcomes would benefit these trials greatly, as would purposeful replication of already-reported significant findings using the same measurement techniques. Otherwise, it also remains an open question as to how much current findings are driven by e.g., different tool sensitivities.

Taken together, it is very difficult to identify a single trial which arguably addresses all of these concerns. Ghalichi et al. [85] is the largest of those identified (80 subjects randomised), but this ran for 6 weeks and was not blinded. The longest running trials were Whiteley et al. [90] and Adams et al. [83], which took principle measurements over 12 months. Each of these did have modest sample sizes (*N* = 73 and *N* = 67 randomised, respectively), but neither were blinded and as discussed Adams et al. included a wide range of accumulative interventions. This highlights a real gap, wherein a well-powered, long-duration and placebo-controlled trial of either the GFD or GCFD has not yet been conducted. Such an experiment would ideally be run after a community consensus is reached regarding what outcomes should be focused on. Until such a trial is conducted a confident, overall conclusion cannot be made. Currently therefore, the overall pattern of the available literature does not support a proved benefit of the GFD in people with ASD (who do not have a clinical diagnosis of CD).

## 7. Adoption of the GFD and GCFD in ASD

Regardless of there being inconclusive evidence of a benefit to the GFD or GCFD in ASD, adoption of speciality diets is high. Studies assessing this also frequently attempt assessment of possible benefits of the diet primarily via cross-sectional analyses utilising symptom scales/survey responses, or anecdotal reporting from caregivers.

Bowers [94] reported that a majority of ASD referrals to their diet service regarded a suggestion to go on a GCFD (54.1%). Two of these 14 referrals later saw families of the patient report a “transformation” following adoption of the GFD diet (“One family described a 90% improvement and another family described an ‘awakening’ from a different level of consciousness”). A small comparison study [95] of children with ASD who were and were not following the GCFD reported that 7 of 13 children with ASD were already on a GCFD when recruited (outcome measures did not differ significantly from the 6 of 13 who were not on the diet, however parents of all children on the GCFD reported that it had improved symptoms and behaviour). Babinska et al. [20] found 20.7% of children and adolescents with ASD to follow a diet which in some way restricted gluten (either GFD or GCFD); it was not found that the following of speciality diet correlated with GI symptom severity. Another study [96] found that 12% of their cohort of children with ASD consumed a GCFD, with these children also more likely to take supplements and overall showing better intake of nutrients including vitamin E, D and magnesium.

Hopf et al. [97] surveyed parents of children with ASD to identify reasons why they engaged with “complimentary and alternative medicine” (CAM). The GCFD had been used at some point by 54.8% of responders, although this was not rated among the interventions which were perceived as having had the greatest effectiveness (which included sensory integration therapy, melatonin and prescription antifungal medication). A similar study [98] also focused on the use of CAM in children with ASD as measured by caregiver report. Here, the GFD was followed at a lower rate (10% of the whole group), but was still the most common speciality diet followed. Rubenstein et al. [99] found 20.4% of children with ASD had ever used a GFD; those currently engaging with it had started on the suggestion of a medical professional in 50.7% of cases. Self-reported (from caregivers) data which predicted use of the GFD included GI conditions and developmental regression. Another experiment [100] examined all inpatients at a university medical centre, who did not have CD but who followed a GFD, to find predictors as to why in terms of comorbidities. In this, it was observed that having ASD led to an odds ratio of being on the diet of 23.42; by far the highest of all significant conditions reported (the next being irritable bowel syndrome with an OR of 6.16).

Studies which focus more directly on matching parental reporting of dietary practice to behavioural outcomes include Pennesi et al. [101]. Here, reports from 387 parents/caregivers of children with ASD were examined which focused on GI symptoms, suspected food sensitivities and adoption of speciality diets (primarily GCFD). Within these reports statistical effects were noted wherein greater suspicion of GI problems predicted greater improvement in ASD outcomes following adoption of speciality diets. Strict diet engagement was also observed to be significantly related to better outcomes. Another study [102] found no associations between dietary intake (which included measurement of gluten) and GI symptoms. However, these authors compared intake of gluten in grams against study outcome and a critical observation may be that gluten often needs to be eliminated entirely to usually see any benefit.

Some research has also focused more on the motivation in parents of children with ASD to adopt a GFD or GCFD. Marsden et al. [103] noted that parents who adopted these diets for their children with ASD were most influenced by “anticipated regret, positive outcomes and attitude”. Perceived control was also relevant as a factor (with more predicting use of the diet). Tarnowska et al. [104] also investigated a similar question of what influenced parents of children with ASD to purchase GCFD foods. Packing features such as clear labelling that the food was e.g., gluten-free made them more likely to buy, while social issues around following exclusion diets (e.g., going out for a meal) and the expense/limited range of GCFD foods were seen as negative points. A survey study [105] found that approximately three quarters of clinical professionals who care for people with ASD had been asked at some point about the GCFD, while 29.5% of parents reported use of the GCFD specifically. Inadequacies with the knowledgebase regarding the use of speciality diets were noted by respondents.

Adoption of the GFD or GCFD in children with ASD is therefore quite pronounced, with lower estimates starting at 10%, and multiple studies reporting >50%. Motivations to engage with the diet appear to revolve around anticipated regret of negative outcomes should it not be tried, as well as a parent having a higher degree of perceived control. A recurring theme in a number of studies is the anecdotal reporting (e.g., by parents) of improvement in ASD outcomes, which are often isolated in incidence but apparently dramatic in effect. Caregivers and clinicians each highlight that greater understanding of how these diets interact with ASD is required.

## 8. Nutritional Considerations

Limited research has also studied the impact of the GFD or GCFD on the nutritional health of children with ASD. Studies which indicate a positive consequence of following the GFD/GCFD on health include one by Herndon et al. [106]. While this focused on comparisons between (all) children with ASD compared to TD children, a subgroup analysis revealed those with ASD who followed a GCFD had higher vitamin E intake than those who did not. As already discussed, Stewart et al. [96] found following a GCFD led to higher levels of vitamin D, E and magnesium, possibly relating to a higher likelihood of simultaneously using supplements compared to those following a regular diet. Supporting this, another study [107] found that those on a GCFD were far more likely to take vitamin D and calcium supplements; no child who followed the GCFD had a deficiency of 25(OH)D (a marker of bone health), compared to 24% of those on a regular diet who did.

Studies of no or mixed outcomes include one [108] where no difference was found in nutritional intake between children with ASD who did and did not follow a GCFD (although an overall effect of suboptimal intake was noted across the whole cohort). Analysing food diaries, Mari-Bauset et al. [109] also reported mixed outcomes wherein children who followed a GCFD had lower BMI and energy, and lower intake of some nutrients (sodium, calcium, phosphorus and pantothenic acid). Conversely however, they had better intake of fiber, legumes, vegetables and fat.

Studies reporting negative impacts of the diet include Arnold et al. [110], who found a trend for children with ASD who followed the GCFD to have more deficiencies relating to essential amino acids, including tryptophan. This was replicated by another investigation [111] which also detected lower tryptophan in children with ASD compared to TD controls (it was lowest in those with ASD who followed a restricted diet). These authors hypothesised this may lead to a worsening of ASD symptoms.

## 9. Synthesis of Literature and Future Directions for Research

The interest of speciality diets in ASD, and particularly the GCFD, has increased markedly in the last 2 decades both in terms of adoption in the ASD community as well as scientific study. Research does convincingly demonstrate certain effects and associations which justify this. Most notably is a modest comorbidity between CD and ASD. Also shown is physiological evidence of inadequate digestion of gluten in people with ASD, leading to elevated “exorphins”/gluten antibodies around which reasonable hypotheses exist regarding downstream negative consequences on the central nervous system. Whether these associations are because of a unique relationship between CD and ASD or because of a predisposition in people with ASD to have a generally higher rate of autoimmune-like features, is yet to be resolved. Further research which directly examines the effects of these gluten products is needed in people with ASD, while additional studies examining shared genetic predispositions would also be warranted and beneficial. There is also a scarcity of epidemiological research characterising the comorbidity of ASD and CD; while one very well powered study does exist and supports this to be the case replication elsewhere is desirable. The availability of newer gluten-related antibodies (e.g., TG6) and the use of native antigliadin antibodies that are known to be sensitive to the whole spectrum of gluten-related disorders, may provide a good opportunity for further large scale epidemiological studies.

Arguably the greatest gap in current literature relates to the lack of tightly designed trials of the GFD, or GCFD in people with ASD. Those that are currently available suffer from very pronounced heterogeneity regarding the intervention followed, sample size, trial duration, blinding and outcomes measured. There does not yet exist an RCT which combines an at-least modest sample size with a placebo-controlled design over a long duration. This would be highly valuable to address, accepting the limitations of the difficulties of such an intervention (gluten free diet) in the context of what is a behaviourally-complex cohort of patients.

Adoption of the GFD and GCFD appears to be very high amongst people with ASD. An impression is gained of strong anecdotal evidence of a benefit in relevant studies, although statistical associations do not as often bear this out. It is also unclear if any reported behavioural benefits would be because of a direct interaction between physiological gluten/casein-related impacts on the brain, or if engaging with speciality diets simply reduce non-specific GI symptoms and thus improve quality of life in a more general sense.

The nutritional impact of a GCFD on the child with ASD generally seems to be slight, or even associated with improved intake. However, some studies showing deficiencies of certain nutrients highlight the need to still maintain a balanced diet once on a restricted one. Finally, a general observation is the abundance of research which focuses on children. Of the initial papers found in the literature review, 68 (of 79) studied groups which were exclusively children, or mixed children and adolescents. While this is likely an outcome of opportunity and convenience sampling effects, it is nonetheless a strong bias within the available literature and means that generalisation of anything discussed in this review to adults with ASD is difficult.

## 10. Conclusions

This review highlights a modest comorbidity between ASD and CD, and a base of evidence on which reasonable hypotheses may be built to explore if gluten has a generally adverse effect in exacerbating the symptoms and quality of life in children with ASD. However, a negative effect of gluten ingestion in ASD has not been proved. Trials which have sought to demonstrate this are variable in their findings, and suffer from issues with experimental design and execution which means that an overall interpretation cannot yet be made. Efforts should focus on future studies which address limitations detailed here to create an RCT from which confident conclusions can be drawn. As diets which restrict gluten see a very high adoption rate among people with ASD, such further research is certainly warranted.

## Figures and Tables

**Table 1:** A summary of studies which have investigated if gliadin antibodies (AGA) are affected by ASD. *: full text not available.

Citation	Cohort (Describes the ASD Group Unless Otherwise Specified)	Finding (% Where It Was Above an Abnormal Cutoff Given Where Possible)
Cade et al. (2000) [63]	150 children and adolescents	IgG AGA raised (87% of group)
Vojdani et al. (2003; the two publications by Vojdani et al. in 2004 also report gliadin antibodies, but use the same dataset) [37,38,39]	50 patients	IgG (44%)/IgA (46%)/IgM (36%) AGA raised compared to controls
Kawashti et al. (2006) [64] *	30 children	AGA raised (50% of group) compared to controls
Batista et al. (2012) [31]	147 patients	IgG/IgA AGA not different to controls
Lau et al. (2013) [65]	37 children	IgG AGA raised (24.2% of group) compared to controls, and particularly in those with a GI medical history
de Magistris et al., 2013 [66]	162 children	IgG AGA raised (25.3% of group) compared to controls, higher in both those on GFD and regular diets.
Józefczuk et al., 2018 [59]	77 patients	IgG AGA raised (27.3% of group)
Abdel-Maksoud et al. (2020) [67]	66 children	IgA AGA titre lowered compared to controls

**Table 2:** A summary of randomised trials which have in some way included a gluten-free diet as an intervention in treating ASD.

Citation	*N* Randomized & Comment on Groupings	Participants Blinded?	Diet(s) Tested	Duration	Any Main Outcomes Significantly Affected by Intervention?
Gonzalez-Domenech et al., 2020 [80]	*N* = 37; crossover design. Mixed children and adolescents with ASD, without allergies to gluten or casein. Everyone on gluten & casein-containing diet at baseline.	No	GCFD vs. regular diet	12 months (6 months per crossover block)	None; those tested included behavioural/cognitive measures (ERC = III, ATEC & ABC), and urinary beta-casomorphin as a marker of poor digestion of casein.
Piwowarczyk et al., 2020 [81]	*N* = 66; parallel group. Children with ASD, without celiac disease/wheat allergy. 8 week, GFD run-in period before start.	No	GFD vs. regular diet	6 months	None; those tested included behavioural/cognitive measures (ADOS-2, SCQ, ASRS, VABS-2, LIPS), and Rome-III for GI symptoms.
Grimaldi et al., 2018 [82]	*N* = 30; parallel groups. Children with ASD who did not take dietary supplements. Baseline food diaries identified groups who already either followed GCFD or regular diets; randomization to receive the prebiotic mixture happened within these groups.	Yes	GCFD + “B-GOS” prebiotic mixture (vs. GCFD without B-GOS, vs. regular diet with/without B-GOS).	6 weeks	Improvement in behavioural scores (ATEC & AQ) in children on GCFD + the prebiotic mixture (not observed in those on GCFD alone). No significant results reported for EQ-SQ or SCAS-P. Physiological changes (urine spectra, faecal samples, were observed in response to the prebiotic mixture, both across dietary groups and between them.
Adams et al., 2018 [83]	*N* = 67; parallel groups. Children and adults with ASD. 2 month run-in of no special diet or supplements.	No	Various interventions, added accumulatively (vs. no diet/modifications). At the end of the trial, interventions included GCFD (for 155 days) + supplementation of vitamins, minerals, essential fatty acids, carnitine, digestive enzymes & taking of Epsom salt baths.	12 months	Improvement in behavioural/intellectual scores (RIAS non-verbal IQ, CARS, SAS Pro, VABS-II, PDDBI Composite, ATEC, ABC, SRS & SSP) Improvement in GI symptoms (measured by 6-GSI). Some changes to complete blood count and blood chemistry panel markers, fatty acid profile, vitamin levels, RBC elements, homocysteine, l-carnitine No changes in handgrip strength or C-reactive protein
El-Rashidy et al., 2017 [84]	*N* = 45; 3 parallel groups (2 dietary interventions, and a controls). Children with ASD	No	GCFD vs. ketogenic vs. regular diet	6 months	Improvement in behavioural/intellectual scores (CARS, ATEC); in both GCFD and ketogenic groups. Degree of change was not sig. different between these groups, but each appear markedly larger than change observed in the control group (this specific comparison does not appear to have been statistically evaluated)
Ghalichi et al., 2016 [85]	*N* = 80; parallel groups. Children and adolescents with ASD, not following any special diets.	No	GFD vs. regular diet	6 weeks	Improvement in behavioural scores (GARS-2). Improvement in GI symptoms (ROME III)
Hyman et al., 2016 [86]	*N* = 14; crossover design. Children with ASD, without celiac disease or wheat/milk allergy. 6 week run-in period of GCFD.	Yes	GFD vs. CFD vs. GCFD vs. regular diet.	12 weeks (Alternating diets delivered in “blocks” where every participant did each diet one week at a time. This was repeated 3 times, totalling 12 weeks)	None; those tested included behavioural scales (CARSA, RRLRS) and physiological scales (Bristol Stool Scale).
Pusponegoro et al., 2015 [87]	*N* = 74; parallel groups. Children with ASD, with high levels of urinary I-FABP excretion (indicating heightened intestinal permeability)	Yes	GCFD vs. regular diet	1 week	No change in behavioural outcomes (AWPC) Worsening of gastrointestinal symptoms; significant in within-group analysis, but change in this measure was not different between groups. No change in urinary I-FABP
Navarro et al., 2015 [88]	*N* = 12; parallel groups. Children with ASD, without celiac disease or food allergies. 2 week GCFD run-in period.	Yes	GCFD vs. regular diet	4 weeks	None; formal statistics generally avoided due to small sample size, but trends were generally absent in all outcomes which included behavioural/intellectual measures (CPRS-R, ABC) and physiological measures (lactulose/mannitol recovery ratio for intestinal permeability, or GI symptoms on a non-validated questionnaire)
Johnson et al., 2011 [89]	*N* = 22; parallel groups. Children with ASD.	No	GCFD vs regular diet	3 months	None; those tested included behavioural scales (CBC, MSEL, blinded observations) and physiological measurements (likert scales RE constipation etc.). Isolated sub-scores of MSEL & CBC were sig., though authors note no consistent pattern and reject them
Whiteley et al., 2010 [90]	*N* = 73; parallel groups. Children with ASD	No	GCFD vs. regular diet	24 months; interim analyses at 8 and 12 months would reassign regular diet participants to receive GCFD for the remainder, if sufficient improvement was observed in GCFD group.	Improvement in behavioural/intellectual outcomes (ADOS, GARS, VABS), no change in ADHD-IV at the 8 month analysis; the control group was added to the diet at 12 months making 24 month data un-comparable.
Elder et al., 2006 [91]	*N* = 13; crossover design. Children and adolescents with ASD, without celiac disease.	Yes	GCFD vs. regular diet	12 weeks (6 weeks per crossover block)	None; those tested included behavioural/intellectual measures (CARS, ECO and observation of in-home behaviour). Authors do note parental reports indicating potential improvements in individual children when on the diet. Parents otherwise performed poorly at guessing which period of time they had been given the GCFD foodstuffs.
Knivsberg et al., 2002 [79]	*N* = 20; parallel groups Children with ASD in additional to abnormal urinary peptides.	No	GCFD vs regular diet	12 months	Improvement in behavioural and intellectual outcomes (DIPAB, LIPS, ITPA, Reynells spraktest, MABC)

Findings described in the final column relate to any analysis which indicates with statistical significance that the intervention affected an outcome. 6-GSI; 6-Item Gastrointestinal Symptom Index, ABC; Abberant Behaviour Checklist, ADHD-IV; Attention-Deficit Hyperactivity Disorder—IV rating scale, ADOS; Autism Diagnostic Observation Schedule, AQ; Autism Spectrum Quotient, ASD; Autistic Spectrum Disorder, ASRS; Autism Spectrum Rating Scale, ATEC; Autism Treatment Evaluation Checklist, AWPC; Approach Withdrawal Problems Composite (a subset of the PDD-BI), CARS; Childhood Autism Rating Scale, CARSA; Conners Abbreviated Rating Scale and Actigraphy, CBC; Child Behavior Checklist, CPRS-R; Connor’s Parent Rating Scale-Revised, DIPAB; Diagnose af Psykotisk Atfærd hos Børn, ECO; Ecological Communication Orientation, ERC-III; The Behavioral Summarized Evaluation, EQ-SQ; Empathy and Systemising Quotient, GARS; Gilliam Autism Rating Scale, GI; Gastro-intestinal, ITPA; Illinois Test of Psycholinguistic Abilities, LIPS; Leiter International Performance Scale, MABC; Movement Assessment Battery for Children, MSEL; Mullen Scales of Early Learning AGS edition, PDD-BI; Pervasive Developmental Disorders Behaviour Inventory, RIAS; Reynolds Intellectual Assessment Scales, RRLRS; Ritvo-Freeman Real Life Rating Scales, SAS Pro; Severity of Autism scale-Professional Evaulation, SCAS-P; Spence’s Children Anxiety Scale-Parent version, SCQ; Social Communication Questionnaire, SRS; Social Responsiveness Scale, SSP; Short Sensory Profile, VABS-2; Vineland Adaptive Behavior Scale, Second Edition.
